# 

**Published:** 1909-08-28

**Authors:** 


### BOOKS RECEIVED.

Digby, Long and Co.

"Gates of Brass." By Mrs. Aubrey Richardson.

Eliot Boothroyd.

"Low's Handbook to the Charities of London."

Rebman, Limited.

"The Maniac."

THE

### GRADUATES' MEDICAL SCHOOLS.

DIARY FOR THE WEEK, AUGUST 30 to SEPTEMBER 4.

**THE POST-GRADUATE COLLEGE, West London Hospital, Hammersmith, W.**

**At 10 a.m.**

**August 30 and September 2,** Surgical Registrar, **Demonstration.**

**September 3,** Medical Registrar, **Demonstration.**

**At 12 noon.**

**August 30,** Dr. Bernstein, **Pathological Demonstration.**

**At 12.15 p.m.**

**August 31,** Dr. Pritchard, **Practical Medicine.**

**September 1,** Dr. Grainger Stewart, **Practical Medicine.**

**At 5 p.m.**

**August 31,** Dr. Robinson, **Gynæcological.**

**September 2,** Mr. Bidwell, **Perforation of Gastric and Duodenal Ulcers.**

### PROPOSED JEWISH HOSPITAL FOR LONDON.

A meeting in support of the proposal to establish a Jewish hospital in London was held on the 15th inst. at the Pavilion Theatre, Whitechapel, and was largely attended. Dr. A. Gaster, who presided, said he deplored the absence of a Jewish hospital in London, and he could not understand how the Jewish community could exist without such an institution. A sum of £1,700 had been collected towards the object they had in view, and it was a question for them to decide whether they were going to relinquish the scheme because disapproval had been expressed of it. It was further stated at the meeting that a site for the hospital had been provisionally secured at Stepney Green, but before paying a deposit it was intended to hold a conference of the Jewish communities. The extent of the area to be acquired was 22,000 feet, and it had been suggested that if Jewish residents in London would subscribe 10s. per foot, £11,000 might be raised towards the cost of the hospital. If half of the 120,000 Jews resident in East London contributed only 1d. per week it would bring in £250 weekly. Application had been made to the richer members of their community, but no definite answer had been received. A resolution approving of the establishment of the hospital was adopted.

